# Supplementary material for: Non-Linear Pharmacokinetics of Oral Roscovitine (Seliciclib) in Cystic Fibrosis Patients Chronically Infected with Pseudomonas aeruginosa: A Study on Population Pharmacokinetics with Monte Carlo Simulations
Source: Pharmaceutics. 2020 Nov 12;12(11):1087. doi: 10.3390/pharmaceutics12111087 (PMC7696167; doi:10.3390/pharmaceutics12111087)
Supplement: Supplementary file 1 [file pharmaceutics-12-01087-s001.pdf]

pharmaceutics

Supplementary Materials: Non-Linear Pharmacokinetics of Oral Roscovitine (Seliciclib) in Cystic Fibrosis Patients Chronically Infected with *Pseudomonas aeruginosa*: A Study on Population Pharmacokinetics with Monte Carlo Simulations

Cyril Leven, Sacha Schutz, Marie-Pierre Audrezet, Emmanuel Noward, Laurent Meijer and Tristan Montier

1. Chemicals and equipment

1.1. Chemicals and reagents

All chemicals and reagents were analytical grade. References are given below:

Chemicals	Supplier	Reference
Acetic acid	Sigma	27225-M
	VWR	20104.298
Acetone	Carlo Erba	412502
Acetonitrile	Carlo Erba	412412000
	VWR	83639.320
Ammonium acetate	Sigma	A7330
Bidistilled water	Carlo Erba	307586
Isopropanol	Carlo Erba	412422000

The solutions were labeled and stored according to the procedures in force at the Test Facility.

1.2. Consumables

Type	Caracteristics	Supplier	Ref.
Analytical Column	Cortecs C18 100x3mm, 2.7µm	Waters	18007372
Autosampler vials	Polypropylene-300µL	VWR	548-0120
Caps	For vials	VWR	548-0435
	For glass tubes	Dutscher	080154
Flask	Class A Volumetric Glass-5mL	Dutscher	090048
	Amber-15mL		215-2586
Tubes	Glass-12x75mm-5mL	Dutscher	110001
	Polypropylene-1.5mL	Dustcher	033290

1.3. Biological matrix

Human plasma (collected with lithium heparin as anticoagulant) were purchased at Biopredic international (Parc d'activité de la Bretêche, 35760 Saint-Grégoire, France).

1.4. Instrumentation and software

Apparatus	Type	Supplier
Agitator	VX2E	IKA-Werke
Autosampler	Nexera X2 SIL30-AC	Shimadzu
Balance	XP 105 DR	Mettler Toledo
Centrifuge	MIKRO220R	Hettich
	Megafuge™ 1.0R	Heraeus Instrument
Column oven	Prominence CTO-20AC	Shimadzu
Evaporator	TurboVap	Caliper/Biotage
Freezer -80°C	BM515	Froilabo
LC-MS/MS system manager	Analyst 1.6	AB Sciex
pH meter	GLP21	Crison
Pump	Nexera X2 LC30-AD	Shimadzu
Refrigerator	335	Liebherr
Ultrasonic bath	Branson 1200	Bransonic
Vortex	Genie 2	Dutscher
	Vibrax VXR Basic	Janke & Kunkel

2. Assay method

Calibration standards and QC samples were prepared from two different stock solutions.

2.1. Preparation of solutions

2.1.1. Preparation of (R)-Roscovitine stock solution

An amount of (R)-Roscovitine, batch No. N0-MRT0-200-3-16 (5.05 ± 0.02 mg), determined taking into account the total correction factor (1.01), was accurately weighed and dissolved into a 5 mL class A volumetric flask by hand shaking in acetonitrile. If necessary stock solutions was sonicated. The volume was adjusted to 5 mL with acetonitrile giving a parent stock solution at 1000 ng/µL.

The stock solution was stored in amber-15mL flask at 5±4°C and stable for at least 26 days.

2.1.2. Preparation of M3 stock solution

An amount of M3, batch No. N0-M3 (5.25±0.02 mg), determined taking into account the total correction factor (1.05), was accurately weighed and dissolved into a 5 mL class A volumetric flask by hand shaking in acetonitrile. If necessary stock solutions was sonicated. The volume was adjusted to 5 mL with acetonitrile giving a parent stock solution at 1000 ng/µL.

The stock solution was stored in an amber-15mL flask at 5±4°C and stable for at least 26 days.

2.1.3. Preparation of the standard working solutions

The standard working solutions were prepared daily from stock solutions.

Concentration (ng/µL)	Added solution		Added volume of ACN (µL)
	Concentration (ng/µL)	Volume (µL)	
100	1000	150 of each stock solutions	1200
20.0	100	100	400
16.0	100	80.0	420
10.0	100	50.0	450
5.00	10.0	250	250
2.00	10.0	100	400
1.00	10.0	50.0	450
0.600	10.0	30.0	470
0.200	2.00	50.0	450

2.1.4. Preparation of the QC working solutions

The QC working solutions were prepared daily from stock solutions.

Concentration (ng/µL)	Added solution		Added volume of ACN (µL)
	Concentration (ng/µL)	Volume (µL)	
100	1000	150 of each stock solutions	1200
16.0	100	80.0	420
10.0	100	50.0	450
0.600	10.0	30.0	470

2.1.5. Preparation of the internal standard stock solutions

An amount of (R)-Roscovitine-D6, batch No. N0-RoscoD6 (5.15 ±0.01mg), determined taking into account the total correction factor (1.03), was accurately weighed and dissolved into a 5.00 mL class A volumetric flask by hand shaking in acetonitrile. The volume was adjusted to 5.00 mL with acetonitrile giving a parent stock solution at 1000 ng/µL.

The stock solution was stored in an amber-15mL flask at 5±4°C and stable for at least 26 days.

An amount of M3-D6, batch No. N0-M3D6 (5.30 ±0.02mg), determined taking into account the total correction factor (1.06), was accurately weighed and dissolved into a 5.00 mL class A volumetric flask by hand shaking in acetonitrile. The volume was adjusted to 5.00 mL with acetonitrile giving a parent stock solution at 1000 ng/µL.

The stock solution was stored in an amber-15mL flask tube at 5±4°C and stable for at least 26 days.

2.1.6. Preparation of internal standard working solution

The stock solutions were diluted daily in acetonitrile in order to obtain a final concentration at 1.00 ng/µL.

2.1.7. Preparation of pH3 buffer

pH 3 buffer was prepared by dissolving around 385 mg of ammonium acetate and diluting 9.28 mL of acetic acid (>99%) with bidistilled water in a 1 L class A volumetric flask. The pH was measured at each buffer preparation.

2.2. Preparation of the calibration standards and QC samples

2.2.1. Calibration standards

A hundred µL of blank matrix were spiked with 5.00 µL of working solution. This preparation, made daily, is detailed in the following table:

Concentration of calibration samples (ng/mL)	Blank matrix (µL)	Added working solution	
		Concentration (ng/µL)	Volume (µL)
**10.0**	100	0.200	5.00
30.0	100	0.600	5.00
50.0	100	1.00	5.00
100	100	2.00	5.00
250	100	5.00	5.00
500	100	10.0	5.00
800	100	16.0	5.00
1000	100	20.0	5.00

2.2.2. Quality control samples

QC samples were prepared daily in duplicate at three concentration levels (30.0, 500 and 800 ng/mL) as described below:

Concentration of calibration samples (ng/mL)	Blank matrix (µL)	Added working solution	
		Concentration (ng/µL)	Volume (µL)
**30.0**	100	0.600	5.00
500	100	10.0	5.00
800	100	16.0	5.00

2.3. Sample preparation

Calibration standards and QC samples were prepared daily. Samples were left to thaw at room temperature for approximately 30 minutes. They were shaken and if necessary, they were centrifuged during three minutes at 4000 rpm (2890g) before sampling.

A portion of each sample (100 µL) was transferred into a microtube. Ten 10.0 µL of the internal standard working solution were added. Samples were then diluted to obtain a constant volume: 5.00 µL of acetonitrile were added to samples and blank matrix spiked with IS, 15.0 µL of acetonitrile were added to blank sample. Samples were then prepared by deproteinization. 500 µL of acetonitrile were added. The tubes were shaken and were centrifuged at 13000 rpm (16000 g) for ten minutes at 4°C. The supernatant was transferred into a glass tube and evaporated under a stream of pure nitrogen at approximately 40°C during 15 minutes. The residue was dissolved in 400 µL of injection solvent and transferred into polypropylene vials. Before injection, vials were centrifuged 3 min at 4000 rpm (2890g).

Chromatography conditions

**Mobile phase**	**pH3 buffer/Acetonitrile (7/3,V/V)**
Elution mode	Isocratic
Analytical column	Cortecs C18, 100x3 mm, 2.7 µm
Injection solvent	Bidistilled water /acetonitrile (7/3,V/V)
Flow rate	1.00mL/min
Injected volume	5.00 µL
Autosampler temperature	+5°C
Oven temperature	+40°C
Needle wash liquid 1	Bidistilled water/acetonitrile (70/30)
Needle wash liquid 2	Acetonitrile/Isopropanol/Acetone (4/4/2)

2.4. Mass spectrometry conditions

The compounds were ionized using turbo V ionization source and detected using the multiple reaction monitoring (MRM) scan type.

Nitrogen was used as nebulizer gas and for collisionally activated dissociation in the collision cell.

Ionisation conditions:

**Parameter**	**Value**
Polarity	positive
Collision gas pressure	10 psi
Curtain gas pressure	40 psi
Ion source Gas 1	50 psi
Ion source Gas 2	50 psi
Temperature	700°C
Ionspray voltage	5500 V

Scan parameters:

Product	Q1 mass (amu)	Q3 mass (amu)	Time (ms)	DP (volts)	EP (volts)	CE (volts)	CXP (volts)
(R)-Roscovitine	355.3	233.1	100	111	10	39	14
	355.3	91.2	100	111	10	67	6
(R)-Roscovitine-D_6	361.3	239.2	100	116	10	41	20
	361.3	91.1	100	116	10	77	6
M3	369.3	323.3	100	111	10	33	8
	369.3	91.2	100	111	10	75	6
M3-D_6	375.3	329.3	100	96	10	33	8
	375.3	91.1	100	96	10	75	6

Resolution Q1: unit
Resolution Q3: unit
The first presented MS/MS transitions for analyte and IS were used for the quantification.

2.5. Data acquisition and calculations

HPLC system, data acquisition, data representation and post-acquisition quantitative analysis were carried out with the LC-MS/MS system manager and integration software of AB Sciex: Analyst TM.1.6

© 2020 by the authors. Licensee MDPI, Basel, Switzerland. This article is an open access article distributed under the terms and conditions of the Creative Commons Attribution (CC BY) license (http://creativecommons.org/licenses/by/4.0/).
